# Improvements of thermal and mechanical properties of achira starch/chitosan/clay nanocomposite films

**DOI:** 10.1016/j.heliyon.2023.e16782

**Published:** 2023-05-27

**Authors:** Rocio Yaneli Aguirre-Loredo, Abril Fonseca-García, Heidy Lorena Calambas, Alejandra Salazar-Arango, Carolina Caicedo

**Affiliations:** a Centro de Investigación en Química Aplicada (CIQA), Blvd. Enrique Reyna Hermosillo 140, Saltillo, Coahuila 25294, Mexico; b Investigadoras por México CONACYT-CIQA, Blvd. Enrique Reyna Hermosillo 140, Saltillo, Coahuila 25294, Mexico; c Grupo de Investigación en Desarrollo de Materials y Productos, Centro Nacional de Asistencia Técnica a la Industria (ASTIN), SENA, Cali, 760003, Colombia; d Facultad de Ingeniería, Unidad Central del Valle del Cauca (UCEVA), Carrera 17a 48-144, Tuluá 763022, Colombia

**Keywords:** Biodegradable films, Biopolymers, Nanoclay, Ultrasonication, Nanomaterials, Thermoplastic starch

## Abstract

Thermoplastic biofilms were developed from achira starch, chitosan and nanoclays using the solvent-casting method. To obtain the filmogenic solutions, different sonication times (0, 10, 20 and 30 min) were considered in order to evaluate the incidence of this parameter on the chemical and physico-mechanical properties of the bionanocomposite films. The chemical analysis using FTIR spectroscopy showed strong intermolecular interactions between the components with increasing sonication times. The results for tensile strength and elongation were satisfactory for films with 20 min of sonication with increases of 154% and 161%, respectively. Morphological analysis showed greater homogeneity, while thermal analysis showed that sonication favoured the plasticization process and thus, the production of homogeneous materials. The water absorption and wettability tests showed less hydrophilic materials allowing these new materials to be considered for use as coatings or packaging for the food sector.

## Graphical abstract

**Figure:**
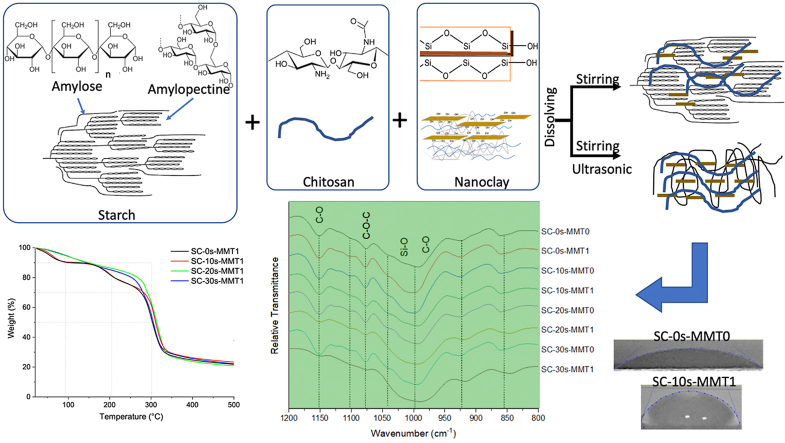

## 1 Introduction

Mankind cannot imagine its daily growth and development without the use of plastics. Plastic is a material that has revolutionized the world thanks to all the advantages and benefits it offers. Plastics began to be derived from the petrochemical industry, bringing versatility to many areas of industrial development. Their use in sectors such as the automotive industry has allowed a significant reduction in vehicle weight and fuel consumption; in the medical sector, it has allowed the creation of new devices that can be used only once, are sterile and resistant, improving the health and safety of patients [1]. In the food area, it helped reduce food loss and waste, increasing its useful life, increasing marketing chains, and obtaining lighter packaging, among other things [2]. However, despite all the good that synthetic plastic brings with it, its slow degradation and widespread use has created an environmental problem that affects almost the entire world. It is currently estimated that plastic consumption exceeds 320 million tons per year, being the last decade when more plastic has been generated [3]. As a result, scientific institutions worldwide have set out to find solutions that reduce plastic pollution, proposing various transformation methods, as well as the replacement of synthetic-based polymers with others that are more environmentally friendly and that biodegrade without harming the environment when discarded. For the development of these sustainable materials, the use of biobased polymers has been evaluated, that is, obtained from natural sources, such as starch, gelatin, chitosan, and cellulose, among others, which due to their natural origin, could be incorporated into the environment without contaminating it and in a much shorter time. These materials have shown some favourable functional properties. However, they were unable to match the mechanical and barrier performance of synthetically derived plastics.

Starch is a biopolymer widely distributed by nature and easily accessible due to its low cost. This vegetable polysaccharide is found in cereals, seeds, legumes and tubers. This last source, with the exception of conventional sources such as potatoes and cassava, is very useful as it does not compete with the food sector. Achira flour is a root that contains between 73 and 85% starch, and at least four ecotypes are recognized: native, green, black and purple in some countries of South America and the Asian continent. These ecotypes according to the agro-ecological zone [4]. The native achira originally from Huila, originally used as an ingredient in baking, has also been studied to propose an alternative biodegradable material [5,6]. The ratio of biopolymers in these achira starches can vary between 20 and 38% amylose and 80-62% amylopectin. It has been concluded that starch-based materials are very susceptible to water damage and weak strength. To solve some of the deficiencies of starch-based materials, it has been sought to mix starch with some other biopolymers such as polyvinyl alcohol (PVOH), proteins, and chitosan, as well as other additives that improve their performance and applicability properties and are also approved for use in the food area. Another biopolymer is chitosan, a polysaccharide derived from chitin, the second most abundant polysaccharide in nature after cellulose [7]. This is the main component of the exoskeleton of crustaceans, mollusks, and arthropods, as well as the cell wall of some fungi. This biopolymer has been shown to have antimicrobial and antifungal capacity, an essential quality in the design of packaging materials and coatings [4]. Chitosan has been studied in a blends with starch looking for synergy between mechanical and surface properties (wettability) that allow reducing the brittleness in the films and hydrophilicity, respectively [8,9]. Another promising strategy relates to the incorporation of reinforcements. These enhance the properties of the materials, which depend on the nature, size, shape, orientation, distribution and concentration of the reinforcement on the matrix. Several studies have shown that the use of nanometric-sized reinforcements allows a better cost-benefit ratio, as minimal amounts (>1%) are required to achieve high functionality and/or performance in the material, supported by the matrix-reinforcement interaction. Among these nano additives, metallic nanoparticles [10], nanofibers [11,12] and nanoclays [13,14]. An important parameter that can define the properties of biobased materials is the method of preparation and incorporation of the additives [15]. Conventional techniques for processing plastic materials, such as thermoforming, casting, or melt mixing, have shown that they do not achieve complete exfoliation of nanoclays, so they interact with the polymeric matrix only in a non-homogeneous intercalated mode [16,17]. Romero-Bastida, Bello-Pérez [15] found in starch films made by the casting method, the order in which the components are added, in this case, the plasticizer glycerol and montmorillonite nanoclay (MMT), significantly affects the behavior of the resulting materials. MMT is a safe and bioinert clay for use in films and packaging that will be in contact with food and drugs—making it an important additive for improving the barrier capacity against gases, such as metal carriers and compounds with antimicrobial activity [18,19]. MMT has shown very low cytotoxicity in cells in in vitro studies, which is related to the concentration of MMT used. The reduction in cell viability could be related to the blockage of channels in the cell membrane when the concentration of this nanomaterial is very high. However, for the development of packaging materials, the proportions of MMT in the formulations are very low, ranging from 1 to 15% (w/w) [15,20]. It has been reported that using technologies such as high-intensity ultrasound can improve the mechanical properties of films made from quinoa-chitosan proteins because it promotes better incorporation of the nanoparticles and favours the cross-linking process of the polymeric chains promoted by the additive used (transglutaminase) [21]. In developing chitosan nanocomposites, the ultrasonic treatment produced a more homogeneous dispersion of silver nanoparticles and carbon nanotubes, increasing nanocomposites' mechanical strength [22]. Likewise, the structure of the MMT nanoclay can be modified and oriented in pectin film-forming solutions by processes such as fluidization [23], extrusion [24]. It has also been shown that the sonication process is necessary to achieve a good dispersion of MMT nanoclay in starch-PVOH polymeric matrices [13]. Ultrasonication has also proved to be an effective treatment for the formation of polymeric systems with nanoparticles where it is desired to generate a homogeneous and stable solution during storage. In solutions of polysaccharide-selenium nanoparticles, ultrasound treatment improved the stability of the particles, obtaining a homogeneous, translucent solution without particles in precipitation and maintaining these stability characteristics for at least 16 days [25]. A study by Kaur, Kalia [26] show conditions for obtaining films by solvent-casting method of starch-chitosan biopolymers blends that incorporate effective nanoparticles (ZnO and Ag) to control microbial growth and maintaining organoleptic properties of peaches up to 6 days. Another work shows the effect of nanoparticle content on the performance of starch-chitosan biopolymeric films at different ultrasound powers [27]. The effect of cavitation on reactions and mixtures depends on the frequency, power and time of application. Several authors have demonstrated the incidence of the ultrasonic effect in favour of the integration of reinforcements on the matrix, greater dispersion, reduction of agglomerates, miscibility of different components in the matrix in favour of homogeneity. Therefore, this study aimed to evaluate different ultrasound application times in bionanocomposite blends on the structural, mechanical, and thermal properties of films based on achira-chitosan starch and montmorillonite nanoclay. This work can serve as a basis for the ultrasonic processing of biopolymeric mixtures and contribute to the applications of these materials of biodegradable origin in the food and pharmaceutical industries as packaging materials, and edible coatings.

## 2 Materials and methods

### 2.1 Materials

Achira starch (density of 1.59 g/mL) was supplied by Surtialmidon (Huila, Colombia). Chitosan was supplied by the supplier Sigma-Aldrich, low molecular weight, degree of deacetylation >75%. Nanoclay hydrophilic bentonite (density of 600–1100 kg/m^3^, and average particle size ≤25 μm), was supplied by the supplier Sigma-Aldrich. Acetic acid with purity (GC): >99.7% and density of 1.13 g/mL, was supplied by Sigma-Aldrich. Glycerol as plasticizer with a density of 1.26 g/mL, and 99.68% purity) was supplied by Sigma-Aldrich (St. Louis, MO, USA).

### 2.2 Film preparation

**Fig. 1:**
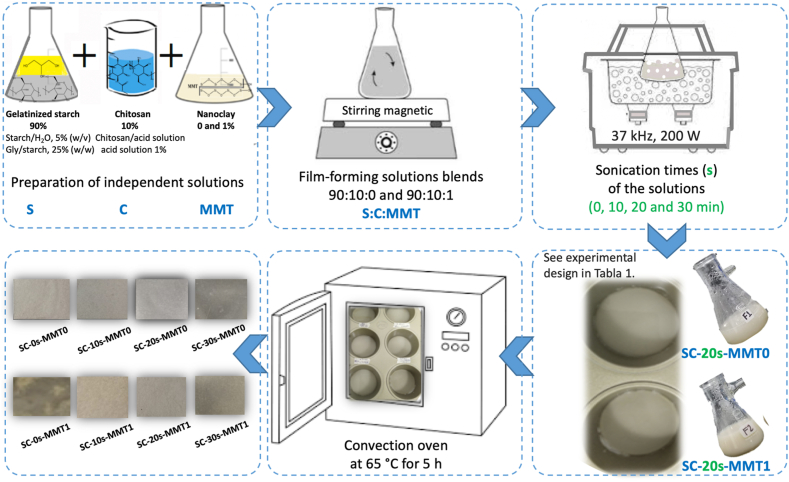
Process for the preparation of bionanocomposite films by the casting method.

A dispersion of achira starch (5% w/v) was prepared in distilled water, to which the plasticizer glycerol was added at 25% (w/w) with respect to starch, the temperature was maintained at 80 °C, 600 rpm for 30 min, conditions similar to those reported in previous studies [13]. Separately, a 10% (w/v) solution of chitosan in 1% (v/v) acetic acid was prepared, and the nanoclay (MMT) was added with constant stirring until it was homogenized entirely with the chitosan solution. Both solutions were mixed in a starch-chitosan ratio of 90:10 (Fig. 1 and Table 1) to obtain the film-forming solutions (FFS) and it was kept under stirring for ∼30 min. FFS without and with nanoclays (MMT) were prepared.

**Table 1:** Composition of film-forming solutions (FFS) and preparation method of starch-chitosan composite films.

Film composition	Starch ratio (%)	Chitosan ratio (%)	MMT nanoclay (% w/w)*	Ultrasonication time (min)
SC-0s-MMT0	90	10	0	0
SC-10s-MMT0	90	10	0	10
SC-20s-MMT0	90	10	0	20
SC-30s-MMT0	90	10	0	30
SC-0s-MMT1	90	10	1	0
SC-10s-MMT1	90	10	1	10
SC-20s-MMT1	90	10	1	20
SC-30s-MMT1	90	10	1	30

Where S: Thermoplastic achira starch, C: Chitosan, MMT: Montmorillonite nanoclay, and s: Ultra sonication process time. *The percentage of added MMT clay was determined based on the total weight of both biopolymers.

MMT 0 and 1%, respectively), and the concentration was determined based on the optimal results of mechanical, wettability and absorbent properties of films obtained in a study previously published by the working group [13]. The FFS were treated with sonication at room temperature (25 °C), and the treatment times varied between 0, 10, 20, and 30 min, as presented in Table 1. The times were taken according to previous reports where it can be seen that after 30 min there are no significant changes to the structure [28]. After the sonication process, the polymer solutions were poured into Teflon moulds and dried in a convection oven at 65 °C for 5 h. The bionanocomposite films were previously conditioned for 48 h at 25 °C and 50% RH before their physical and chemical properties were evaluated.

### 2.3 Characterization

#### 2.3.1 Fourier transformed infrared spectroscopy (FTIR)

The FTIR analysis was performed on the films in attenuated total reflection (ATR) mode [6]. An IR Affinity-1 spectrometer (Shimadzu Corporation, Kyoto, Japan) was used under 16 scans during the analysis. In a range between 600 and 4000 cm^−1^ wavenumber and a resolution of 4 cm^−1^.

#### 2.3.2 Thermal properties

Thermogravimetric analysis (TGA) and differential scanning calorimetry (DSC) of biodegradable films with and without nanocomposites were performed to assess thermal stability and their respective thermal transitions [6]. A TGA/DSC 2 STAR System thermogravimetric analyzer (Mettler Toledo, USA) was used. The analysis conditions were as follows: heating rate: 10 °C.min^−1^, temperature range: 25 °C to 500 °C, inert atmosphere (nitrogen) flow: 50 mL min^−1^, sample amount 10 ± 0.5 mg.

#### 2.3.3 Tensile test

The tensile test was performed to determine the mechanical properties of the materials, using an INSTRON model EMIC 23–50 universal machine (São José dos Pinhais, PR, Brazil) equipped with a 50 kN load. Measurements were made on 6 rectangular specimens for each sample (formulation) with dimensions of a length of 10 cm and wide of 2.5 cm and a thickness variable, at a speed of 1 cm min^−1^ until the sample ruptured, according to the ASTM D882 standard [29] and Fonseca-García, Caicedo [30]. Standard test method for the tensile properties of thin plastic sheeting. ASTM International). The samples were previously conditioned at 23 °C for 48 h. Only materials obtained from ultrasonically treated film-forming solutions were used for this characterization.

#### 2.3.4 Scanning electron microscopy

The surfaces of films were examined and the micrographs were digitally captured using a scanning electron microscope (SEM) JCM 50000 (JEOL, Tokyo, Japan). A voltage of 10 kV was applied, according to the methodology described by Fonseca-García, Caicedo [30]. The samples were coated with a thin layer of gold. Magnifications of 1000x of the fracture surface were taken.

#### 2.3.5 Contact angle

Contact angle measurements were made to evaluate the wettability of the materials when exposed to normal environmental conditions using distilled water. Contact angle measurements were made using a goniometer (Ramé-Hart Instrument Co., model 250, NJ, USA) at room temperature, following the methodology from Caicedo, Aguirre Loredo [6]. A 20 μL drop of distilled water was placed on the surface of the polymer samples. After a stabilization period of 60 s, the image was recorded, and the contact angle was measured using the free software ImageJ. This measurement was performed in triplicate.

#### 2.3.6 Water absorption

The 2 × 2 cm films were immersed in liquid water for 6 h and the change in weight of the composite material is recorded [31]. ASTM D570 standard was adopted to perform the water absorption tests. Percentage weight gained (*%W*) by the composite samples due to water absorption was calculated using Equation (1).

(1) $$\%W=\frac{W_{t}-W_{0}}{W_{0}}x100$$

Where, *W*_*t*_ is the weight of specimen after 6 h of immersion (equilibrium time for the bionanocomposites), and *W*_*0*_ is the weight of dried specimens. This determination was performed in triplicate.

### 2.4 Statistical analysis

Means and standard deviations are presented, and analysis of variance (ANOVA) was used to compare mean differences in film characteristics. In addition, the comparison of means was performed using Tukey's test at a significance level of 0.05. All statistical analyses were performed using IBM® SPSS® Statistics 25 software.

## 3 Results and discussions

### 3.1 FTIR analysis of film

**Fig. 2:**
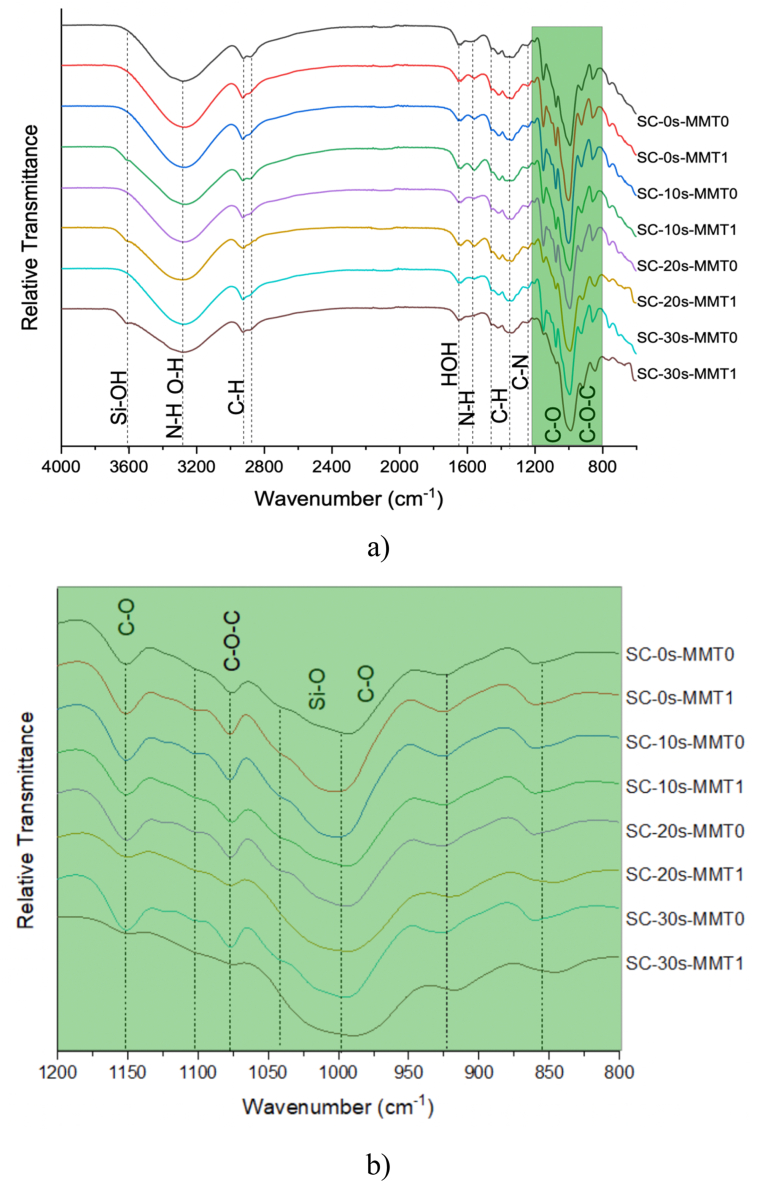
FTIR-ATR spectra of starch-chitosan nanocomposite films. a) 4000-600 cm^−1^, b) 1200-800 cm^−1^.

Fig. 2a shows the spectra of bionanopolymeric films. On the one hand, starch showed characteristic bands at 3320 cm ^−1^, due to O–H stretching, this absorption band is strong and broad and these bands overlap with the –NH stretching vibration of chitosan and glycerol hydroxyls. At wave numbers 2924 cm^−1^ and 2887 cm^−1^, vibrations of the methylene (CH_2_) groups were observed due to symmetric and asymmetric stretching, respectively. At 1646 cm^−1^, the bending vibration of OH (water) and the C=O stretching band of the amide group that appears in chitosan with the same wave number [32], as well as the bending for –NH_2_ at 1554 cm^−1^. The deformations of CH and CH_2_ are evident at 1412 cm^−1^, and the bending at 1378 cm^−1^ for –CH_2_. Around 1242 cm^−1^, O–H (bending) vibrations are present. In the ethers zone between 1150 cm^−1^ and 925 cm^−1^ (see Fig. 2b) (C–O stretching and O–H bending), similarly, In the ether zone between 1150 cm^−1^ and 925 cm^−1^ (see Fig. 2b) (C–O stretching and O–H bending). The posterior region between 997 and 706 cm^−1^ presents the vibrational modes of the pyranose ring [33]. At 851 cm^−1^ vibrations of the H atoms are observed. Other flexures are observed between 651 cm^−1^ and 600 cm^−1^ [34]. The different functional groups of the polymers and the bentonite allow us to see an exfoliating effect caused by the collapse of the cavitation bubbles generated by ultrasound. A significant decrease is evident in the bands at 1075 cm^−1^, associated with *v* (C–O) and 1152 cm^−1^ equivalent to *v* (C–O–C) glycosidic. This decrease is due to strong interactions generated by –OH with oxygen attached to carbons. Therefore, the –OH band is reduced due to mobility limitations or restrictions [32]. Similarly, the vibration of the terminal silanol groups (Si–OH) in MMT nanoclays is observed at 3615 cm^−1^ in SC-10s-MMT1, SC-20s-MMT1 and SC-30s-MMT1 films, all of the above understand different times of sonication. On the other hand, in the homologues that were not sonicated, these groups remain highly affected in their non-separated laminar structure, which is why they are not visible in the spectrum [35].

### 3.2 Thermogravimetric analysis (TGA) of films

**Fig. 3:**
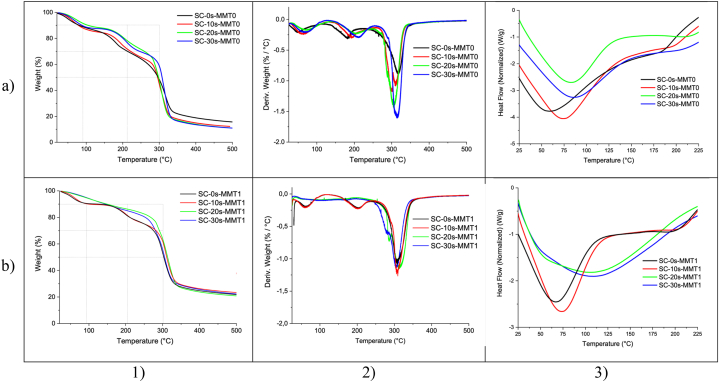
Thermal analysis of starch-chitosan films a) without and b) with nanoclay. 1) Thermogravimetric Analysis (TGA), 2) Difference Thermogravimetry (DTG), and 3) Differential Scanning Calorimetry (DSC).

The thermogravimetric analysis curves of the polymeric biofilms present three cascading thermal events that coincide with T_10_, T_30_, and T_50_, equivalent to weight losses of 10%, 30%, and 50%, respectively, indicated in the grid of Figs. 3–1a and Figs. 3–1b. The T_10_ presents low variations around 8 °C. T_30_ shows an increase in stability between the different sonication times and the blank (the non-sonicated sample) with variations between 8.5 °C and 44.2 °C. Finally, the T_50_ showed a trend with a gradual increase until it reached 10 °C. With regard to the maximum degradation temperature it does not materialize in an increase, however, the variation oscillates between 3.7 °C and 6.9 °C. Regarding the thermal transitions of the biopolymeric film, a change in the gelatinization temperatures (T_g_) can be evidenced (Fig.s. 3–a and Fig.s. 3–b), which shows that this has not been completed for the starch, and that it modifies as the sonication times increase due to possible microstructural changes at the level of microdomains. Iida et al., in 2008 [36] studied the efficiency in the disintegration of the granule by ultrasound action, due to the acceleration of the depolymerization process separating the amylopectin units and leaching the amylose units. This restructuring is possible because it allows the alignment of polymer chains which then form networks that allow the diffusion of smaller molecules (plasticizers). In the case of chitosan, the sonochemical effect is similar because it has a structure similar to that of polysaccharides [37]. For bionanocomposites there is an important change in chemical stability when integrating nanoclays in residence times under ultrasonic radiation (>10 min). These changes are noticeable by showing only one thermal event corresponding to the temperature and maximum degradation (T_d_), Figs. 3–2a and Figs. 3–2b. Although this does not lead to an increase in T_d_, if it shows complete gelatinization, the nanoclays manage to completely destructure the granule structure due to the mechanical energy that leads to collision and molecular friction [38]. Therefore, an endothermic transition is observed at 104.8 °C and 107.1 °C corresponding to the melting temperature (T_m_) for SC-20s-MMT1 and SC-30s-MMT1, respectively. Similar thermal behavior was obtained in chitosan and starch composites with TiO_2_ and graphene nanoparticles in a study carried out by Liu and Liu [27]. The specific values of the thermal analysis are presented Table 2.

**Table 2:** Summary of thermal analysis values of starch-chitosan composite films.

Film sample	Temperatures (°C)					
	T_10_	T_30_	T_50_	T_d_	T_g_	T_m_
SC-0s-MMT0	81.7	214.3	296.2	316.6	57.0	–
SC-10s-MMT0	78.9	222.7	296.5	311.2	74.1	–
SC-20s-MMT0	89.9	258.5	297.9	309.7	81.8	–
SC-30s-MMT0	88.5	246.6	307.9	312.9	85.8	–
SC-0s-MMT1	93.2	274.8	302.9	309.1	66.9	–
SC-10s-MMT1	109.7	280.1	310.9	307.1	72.7	–
SC-20s-MMT1	149.8	281.9	313.4	315.1	–	104.8
SC-30s-MMT1	145.1	290.9	305.9	309.1	–	107.1

### 3.3 Mechanical properties

The mechanical properties of composite starch-chitosan-montmorillonite nanoclay (MMT) films are shown in Table 3. A significant change in the mechanical performance of the films was observed as a function of the sonication time. In the formulations without nanoclay (referred to as MMT0), it was observed that the ultrasound treatment increased the tensile strength and young's modulus. At the same time, the elongation percentage decreased as the sonication time increased. If the ultrasound treatment is carried out for 30 min, a significant decrease in the material's tensile stress and elongation capacity is promoted. At the same time, the stiffness (Young's modulus) continues to increase.

**Table 3:** Mechanical properties of starch-chitosan (SC) films reinforced with MMT nanoclay and treated with ultrasound during 0, 10, 20 or 30 min).

Film sample	Tensile strength (MPa)	Elongation at break (%)	Young's modulus (MPa)
SC-0s-MMT0	1.72 ± 0.29^a^	15.44 ± 9.9^a^	0.063 ± 0.01^a^
SC-10s-MMT0	1.78 ± 0.32^a^	12.39 ± 9.6^b^	0.081 ± 0.01^b^
SC-20s-MMT0	2.40 ± 0.37^b^	12.16 ± 7.3^b^	0.116 ± 0.02^b^
SC-30s-MMT0	2.63 ± 0.25^b^	10.42 ± 4.2^b^	0.123 ± 0.01^b^
SC-0s-MMT1	2.73 ± 0.30^a^	39.45 ± 10.9^a^	0.179 ± 0.01^a^
SC-10s-MMT1	3.11 ± 0.32^a^	51.30 ± 9.6^b^	0.210 ± 0.01^b^
SC-20s-MMT1	4.21 ± 0.37^b^	63.66 ± 7.3^b^	0.198 ± 0.03^b^
SC-30s-MMT1	3.98 ± 0.25^b^	45.13 ± 4.2^b^	0.283 ± 0.02^b^

Different letters (a, b) in the same column indicate significant differences (*p* < 0.05) between the mixtures with the same nanoclay content but at different times of the ultrasound process. Mean of six replicates ± standard deviation.

Various studies have shown that ultrasonic treatment favors a better dispersion of the additives, reducing the particles' size and generating a narrower distribution. Therefore, ultrasonic treatment is very useful when adding additives of reduced size since materials with smaller particles and higher homogeneity in their distribution will be obtained when using it [25,39]. Ultrasonication, through the effect of acoustic cavitation, generates an excessive but transient pressure, that relaxes the polymeric chains and can modify the structural network of the material. The stress induced by ultrasound loosens the networks of polymeric chains, allowing them to partially unravel more easily [25]. This is when nanomaterials such as the MMT used in this study are inserted into the internal structure of the material and not only in the superficial molecular chains. This process makes it possible to obtain a more homogeneous system with more stable intermolecular bonds, with better-dispersed nanoparticles, and, therefore, materials with better elongation capacity and tensile strength, as observed in the results obtained in this study (Table 3). Short-term ultrasound treatment (10 min) increases the strength of the structural network of mixtures of polysaccharides-nanoparticles; however, when the ultrasound time is prolonged (30 and 60 min), a weakening of the network structure is generated [39]. An effect similar to that observed in this study, where a significant decrease in the mechanical properties (tensile strength and elongation at break) of the materials occurred when the ultrasound treatment was longer than 20 min, even in the formulation without nanoclay (only the elongation behavior).

### 3.4 Scanning electronic microscopy (SEM)

**Fig. 4:**
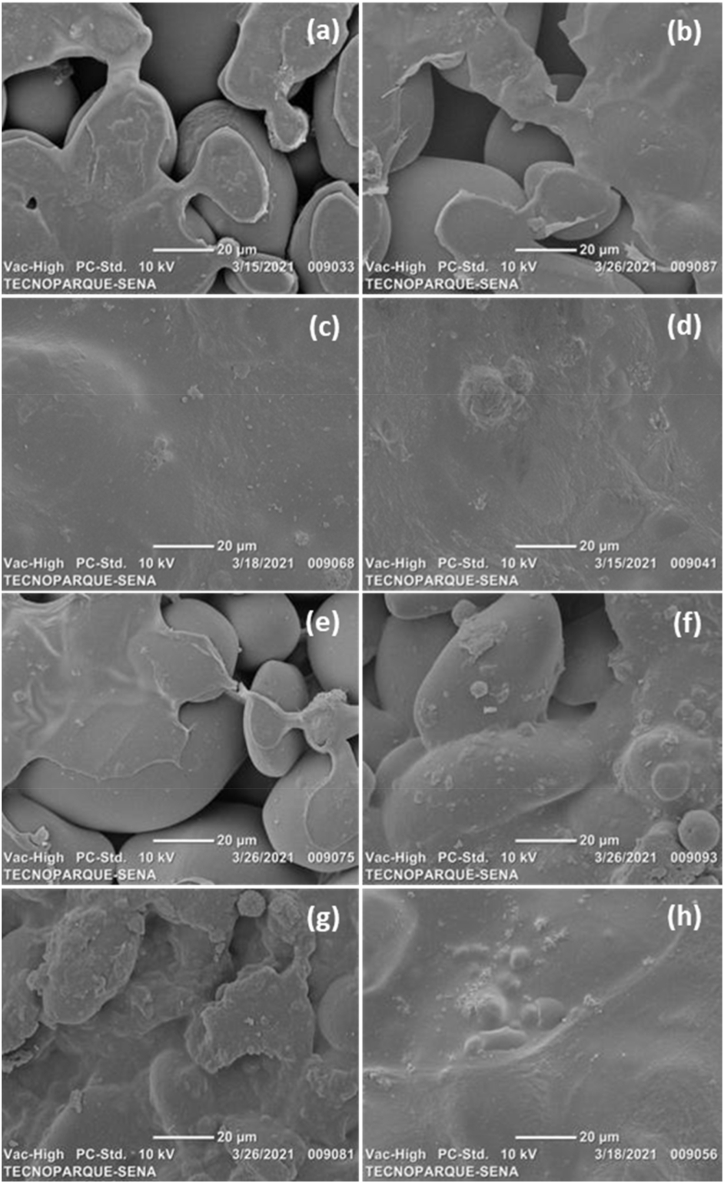
SEM micrographs of starch/chitosan nanocomposites (a) SC-0s-MMT0, (b) SC-10s-MMT0, (c) SC-20s-MMT0, (d) SC-30s-MMT0, (e) SC-0s-MMT1, (f) SC-10s-MMT1, (g) SC-20s-MMT1, and (h) SC-30s-MMT1.

From the images obtained by scanning electron microscopy, it can be observed that the sonication time to which the filmogenic solutions were subjected favoured the destructuring of the achira starch in the films. This process also improved the mixing of the two biopolymers; the sonication caused the chitosan to be better disperse within the achira starch matrix. The achira starch matrix showed relatively good interfacial adhesion, producing smoother or more homogeneous materials as the sonication time was increased. Due to without and with 10 min of sonication, the achira starch granules are visibly present in the material such as shown in Fig. 4a–f. While in Fig. 4c, d, g h the destructuration of achira starch granules is achieved, thus the good interaction among starch, chitosan and/or MMT is done at sonication times of 20 and 30 min. When comparing the microphotographs of the films formulated with and without MMT nanoclay at the longest sonication times (20 and 30 min), it was observed that the addition of nanoclays produces materials with a rougher surface. This result is to be expected since the presence of additives, even of nanometric size, produces surfaces with a less homogeneous or less smooth appearance. The sonication process undoubtedly has an effect on the homogenization of the samples [40], being more evident in the formulations with MMT. The samples without nanoclay (MMT0) present a surface where small particles can be observed; however, there is no phase separation (Fig. 4a–d).

While in the films with nanoclay (MMT1) (Fig. 4e–h), some aggregates are observed, which could be clusters of nanoclays. However, these aggregates decrease in size and show a better dispersion when the film-forming solutions are treated with ultrasound. This result may be a consequence of the cavitation effect, which promotes the exfoliation of the laminar structure of the nanoclays, distributing more efficiently in the starch and chitosan matrix. The same effect was observed by Ashori and Bahrami [41], who obtained nanographene-reinforced films of starch and chitosan by casting (the same method used in this study). The authors found that graphene sheets, when dispersed uniformly, produce an efficient interfacial charge transfer, which is reflected in a uniform stress distribution, marking a significant increase mechanical property of the material. When the sheets of this type of nanometric materials are distributed unidirectionally and stacked, the mechanical performance of the films is poor.

### 3.5 Contact angle

**Fig. 5:**
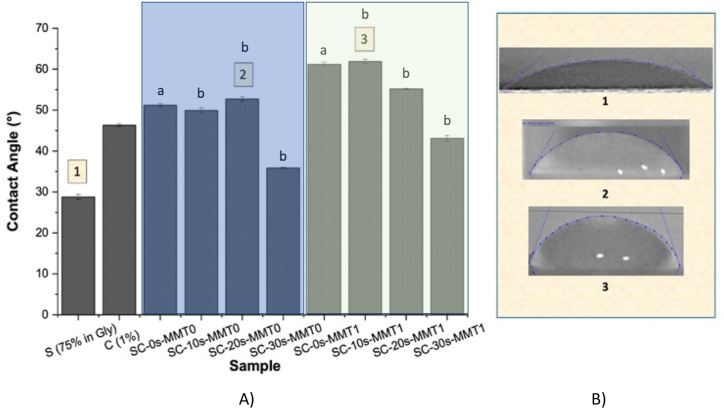
A) Contact angle values obtained by contacting a drop of water on the surface of films based on starch-chitosan (CS) with and without MMT nanoclay obtained from film-forming solutions treated with ultrasound (s) at different times (0, 10, 20, and 30 min). B) Picture of some contact angle measurements.

Contact angle analysis is the appropriate technique to determine the degree of wettability of packaging materials intended for the food area. The affinity of the surface of the material is expressed by measuring the angle between the drop of the solvent used and the polymer on which is placed; this is a function of the time they remain in contact [42]. In this study, distilled water was used as the solvent used since most foods have a high water activity, and it is therefore essential to evaluate the wettability behavior of materials against this liquid is essential. The drop of water was placed on the film samples and left in contact for 60 s, and then the angle formed was determined. For this analysis, films of each polymer alone (starch and chitosan) were evaluated, as well as the mixtures with and without nanoclay presented in Table 1. In Fig. 5, shows the results of the wettability analysis of the starch-chitosan films with and without nanoclays treated with ultrasound.

It was observed that the pure starch films had the smallest contact angle (28°); this is due to the strong interaction of water with starch, which is a biopolymer of a hydrophilic nature. When the starch is mixed with the chitosan, a material with a higher contact angle (>50°) is achieved, which remains slightly unchanged when the filmogenic solutions (without nanoclays) are treated with ultrasound for a maximum period of 20 min. When these polymeric solutions are sonicated for 30 min, the resulting materials increase their hydrophilicity, with contact angles very similar to those of the pure starch film.

Different letters (a, b) in the same column indicate significant differences (*p* < 0.05) between the mixtures with the same nanoclay content but at different times of the ultrasound process. Mean of six replicates ± standard deviation.

In the CS films with MMT, an apparent increase in the contact angle of the material was observed, indicating that its hydrophilic nature was reduced. With the incorporation of nanometric materials, such as CNC, this effect of increasing the contact angle has also been reported in both chitosan and alginate films [43]. However, the effect of ultrasonication on the samples containing clays was very similar effect to that of the blends without clay. Where by increasing the sonication time to 20 and 30 min, a reduction in the contact angle of the materials by approximately 10° is observed. The results suggest that all the films with and without nanoclay are hydrophilic in nature as all the contact angles are <90° [17,44,45]. Considering that MMT is a naturally hydrophilic clay, like starch, it was expected that the resulting materials were expected to maintain their hydrophilic affinity. However, the nanoclay incorporation into films increased the hydrophobic behavior with respect to the similar film but without nanoclay.

### 3.6 Water absorption test

**Fig. 6:**
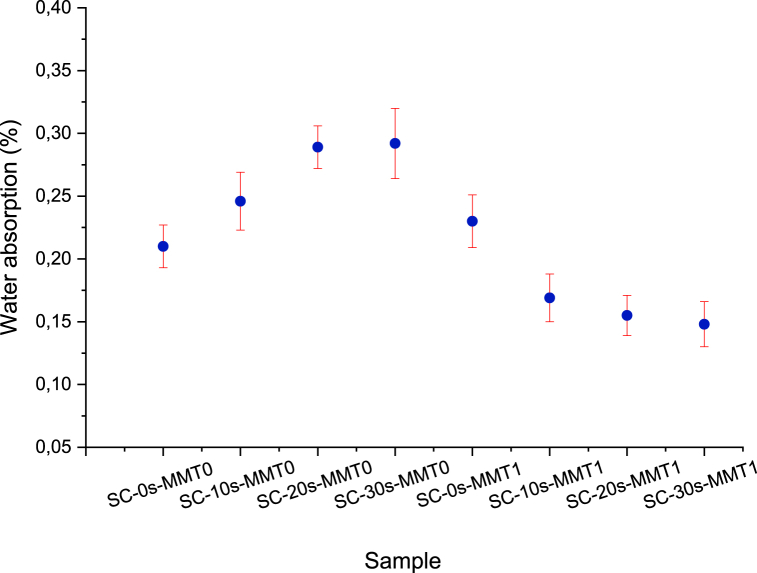
Water absorption of biopolymers and bionanocomposites polymeric films.

The water absorption property of the films was directly proportional to the exposure time to ultrasound (Fig. 6), the above, when it comes to unloaded biopolymeric films. This behavior was due to the hydrophilic nature of the starch associated with a large number of hydroxyl groups present in its chemical structure and the use of glycerol as a plasticizer [46,47]. It should be noted that prolonged times and/or high ultrasound intensities induce chain scission, which allows higher availability of polar functional groups that can generate intermolecular interactions from hydrogen bonds, favoring water solubility [48]. In contrast, bionanocomposite polymeric films produce a barrier showing a significantly lower water absorption capacity due to optimal nanoclay dispersion and starch destructuring. This demonstrates an interaction between polymer chains and intercalation with nanoparticles without inducing nucleation in the materials. These results complement the DSC analysis by showing thermal transitions related to complete gelatinization and, in general, the presence of an amorphous system.

## 4 Conclusions

Bionanocomposite blends of achira starch and chitosan with plasticizer additives and nanofillers processed by casting allow the formation of films with physical properties of interest. The residence time of the bionanocomposite blends under the effect of cavitation due to sonication conditions, proved to be an indispensable variable in the optimization of materials. The strong intermolecular interaction between the OH functional groups present in the polymer matrix and the nanoclay was demostrated using FTIR-ATR. A longer residence time of the solution under sonication favours an increase in the band of the silanol group in the bionanocomposites. The foregoing, due to interlaminar opening of the clay. The repercussion of this effect materializes in the SC-10s-MMT1 and SC-20s-MMT1 mixtures, present themselves as materials with higher thermal stability, gelatinization, and plasticization. While the mechanical performance, contact angle, and water adsorption resistance were also significantly favoured when the sonication time was 10 to 20 min. This is explained by the fact that the ultrasound treatment promoted an adequate dispersion of the MMT clay nanoparticles and their penetration into the polymeric matrix, reducing the formation of granules or aggregates. Composite materials based on starch, chitosan, and nanoclays are a viable alternative for the packaging area in the food sector. Since these materials, in addition to their use as conventional packaging, have a high potential to function as active packaging, it is proposed to investigate the antimicrobial and antifungal properties of these novel materials developed in this study.

## Declarations

### Author contribution statement

Rocio Yaneli Aguirre-Loredo: Conceived and designed the experiments; Analyzed and interpreted the data; Contributed reagents, materials, analysis tools or data; Wrote the paper.

Abril Fonseca-García, Alejandra Salazar: Analyzed and interpreted the data; Contributed reagents, materials, analysis tools or data; Wrote the paper.

Heidy Lorena Calambas: Performed the experiments; Analyzed and interpreted the data; Contributed reagents, materials, analysis tools or data; Wrote the paper.

Carolina Caicedo: Conceived and designed the experiments; Performed the experiments; Analyzed and interpreted the data; Contributed reagents, materials, analysis tools or data; Wrote the paper.

### Data availability statement

Data will be made available on request.

### Data and code availability

The authors declare the transparency of data. Software and programs used in this manuscript have license to be used.

### Supplementary information

Not Applicable.

### Ethical approval

The authors statement that the manuscript it is original and it has not been submitted elsewhere.

## Declaration of competing interest

The authors declare that they have no known competing financial interests or personal relationships that could have appeared to influence the work reported in this paper.
